# Two Phase Modulation of ${\text{NH}}_{4}^{+}$ Entry and Cl^−^/${\text{HCO}}_{3}^{-}$ Exchanger in Submandibular Glands Cells by Dexmedetomidine

**DOI:** 10.3389/fphys.2017.00086

**Published:** 2017-03-01

**Authors:** Minjeong Ji, Chul-Kyu Park, Jin Woo Lee, Kook Yang Park, Kuk Hui Son, Jeong Hee Hong

**Affiliations:** ^1^Department of Physiology, Lee Gil Ya Cancer and Diabetes Institute, College of Medicine, Gachon UniversityIncheon, South Korea; ^2^Department of Molecular Medicine, School of Medicine, Lee Gil Ya Cancer and Diabetes Institute, Gachon UniversityIncheon, South Korea; ^3^Department of Thoracic and Cardiovascular Surgery, Gachon University Gil Medical Center, Gachon UniversityIncheon, South Korea

**Keywords:** dexmedetomidine, secretion, ion transporters, submandibular gland, phosphodiesterase 4

## Abstract

Dexmedetomidine (Dex), a highly selective α2-adrenoceptor agonist, attenuates inflammatory responses induced by lipopolysaccharide (LPS) and induces sedative and analgesic effects. Administration of Dex also reduces salivary secretion in human subjects and inhibits osmotic water permeability in rat cortical collecting ducts. However, little is known about the mechanisms underlying the effects of Dex on salivary glands fluid secretion. We demonstrated the α2-adrenoceptor expression in the basolateral membrane of mouse submandibular glands (SMG). To investigate fluid secretion upon treatment with Dex, we studied the effects of Dex on the activity of Na^+^-K^+^-2Cl^−^ cotransporter1 (NKCC1) and Cl^−^/${\text{HCO}}_{3}^{-}$ exchange (CBE), and on downstream pro-inflammatory cytokine expression in isolated primary mouse SMG cells. Dex acutely increased CBE activity and NKCC1-mediated and independent ${\text{NH}}_{4}^{+}$ entry in SMG duct cells, and enhanced ductal fluid secretion in a sealed duct system. Dex showed differential effects on cholinergic/adrenergic stimulations and inflammatory mediators, histamine, and LPS, stimulations-induced Ca^2+^ in mouse SMG cells. Both, histamine- and LPS-induced intracellular Ca^2+^ increases were inhibited by Dex, whereas carbachol-stimulated Ca^2+^ signals were not. Long-lasting (2 h) treatment with Dex reduced CBE activity in SMG and in human submandibular glands (HSG) cells. Moreover, when isolated SMG cells were stimulated with Dex for 2 h, phosphodiesterase 4D (PDE4D) expression was enhanced. These results confirm the anti-inflammatory properties of Dex on LPS-mediated signaling. Further, Dex also inhibited mRNA expression of interleukin-6 and NADPH oxidase 4. The present study also showed that α2-adrenoceptor activation by Dex reduces salivary glands fluid secretion by increasing PDE4D expression, and subsequently reducing the concentration of cAMP. These findings reveal an interaction between the α2-adrenoceptor and PDE4D, which should be considered when using α2-adrenoceptor agonists as sedative or analgesics.

## Introduction

Dexmedetomidine (Dex) is a selective α2-adrenoceptor agonist with sedative, analgesic, anxiolytic, and hemodynamic stabilizing properties and has protective effects on systemic inflammation by attenuating oxidative stress (Kiliç et al., 2012; Shen et al., 2013). Several studies have shown that Dex benefits various organs, including the heart (Okada et al., 2007; Kocoglu et al., 2008), kidney (Liu et al., 2016), intestine (Sun et al., 2015), and brain (Engelhard et al., 2002). Moreover, Dex exerts its protective effects by inhibiting interleukin (IL)-1β-induced IL-6 synthesis (Tanabe et al., 2014) and by reducing of lipopolysaccharide (LPS)-induced production of pro-inflammatory cytokines, such as IL-1β and IL-6 (Peng et al., 2013). Previously, we demonstrated that Dex attenuated histamine-induced Ca^2+^ increase and IL-6 expression in human salivary glands (HSG) cells (Yang and Hong, 2015). Although, Dex reduces inflammation, there are side effects associated with it, such as reduced salivary secretion (Karhuvaara et al., 1991; Bischoff and Kochs, 1993) and prevented hypersecretion (Marks et al., 2010). Monoxidine, a α2-adrenoceptor and imidazole agonist, mediates salivary glands vasoconstriction leading to hyposalivation (Moreira et al., 2013). Activation of α2-adrenoceptors inhibits adenylate cyclase, thereby reducing cAMP accumulation and cAMP-dependent protein kinase signaling (Rabin et al., 1997).

Fluid secretion and transport are regulated by multiple inputs, such as neurotransmitters-induced Ca^2+^ and cAMP levels in acinar cells (Lee et al., 2012). However, little is known about the regulatory effects of Dex on salivary glands function. Elevation of Ca^2+^ levels by neuronal inputs, such as acetylcholine, stimulate fluid secretion by increasing luminal efflux of Cl^−^ through Ca^2+^-activated Cl^−^ channels. Cl^−^ influx into the cytoplasm occurs against its electrochemical gradient via the basolateral Na^+^-K^+^-2Cl^−^ co-transporter 1 (NKCC1) and via equivalent activities of the Na^+^-H^+^ exchanger and the Cl^−^-${\text{HCO}}_{3}^{-}$ exchanger (Lee et al., 2012). ${\text{HCO}}_{3}^{-}$ transport in the salivary glands requires basolateral ${\text{HCO}}_{3}^{-}$ input via the Na^+^-${\text{HCO}}_{3}^{-}$ co-transporter and subsequent luminal ${\text{HCO}}_{3}^{-}$ output through solute carrier transport 26 (SLC26) superfamily proteins, such as SLC26A6 (Lee et al., 2012; Jeong and Hong, 2016). The Ca^2+^ and cAMP signaling pathways synergistically regulate fluid and ${\text{HCO}}_{3}^{-}$ secretion as a common signaling synergism (Lee et al., 2012; Hong et al., 2014). Synergistic regulation of Ca^2+^ and cAMP resulted in the activation of ion transporters, which induce changes in intracellular pH (pH_i_; Park et al., 2013)

Phosphodiesterase (PDE) reduces intracellular cAMP and cGMP concentrations (Omori and Kotera, 2007). In the salivary glands, PDE4 is involved in the release of amylase from parotid acinar cells (Satoh et al., 2009). The PDE4 inhibitor rolipram regulates ductal ${\text{HCO}}_{3}^{-}$ secretion and confers a protective effect on lipopolysaccharide (LPS)-induced Ca^2+^ signaling and inflammatory cytokine expression (Lee et al., 2016). Although, it has been suggested that Dex is involved in cAMP-mediated cellular events (Rouch et al., 1997), it is unknown whether Dex-modulation of cAMP levels plays a role in ion transporter activity during salivary secretion or whether Dex modulates expression of cAMP-dependent PDE4 of the salivary glands.

LPS is a characteristic component of the outer membrane of Gram-negative bacteria, and LPS triggers innate immune responses to wide range of pathogens by binding to Toll-like receptor 4 (TLR4; Dauphinee and Karsan, 2006). Enhanced expression of TLRs in salivary tissue is associated with progression of inflammatory reactions in autoimmune diseases, such as Sjögren's syndrome (Spachidou et al., 2007). Moreover, TLR activation triggered by LPS enhances oxidative signaling (Liu et al., 2015). Oxidative stress is closely related to inflammation, which is a risk factor in oral disease. It is important that patients with chronic kidney disease report oral complications, such as dry mouth (Ersson et al., 2011), as it may be an indicator of salivary glands dysfunction due to oxidative stress. The regulatory effects of Dex on ion transporters and on fluid secretion from the salivary glands during LPS exposure remain unclear.

We hypothesized that Dex could protect against LPS-induced inflammatory signaling. Therefore, we investigated the anti-inflammatory properties of Dex associated with Ca^2+^ signaling in salivary glands acini and ductal cells upon exposure to *Porphyromonas gingivalis* LPS. We also determined whether Dex has regulatory effects on the ion transporters that mediate fluid secretion and on the cAMP-PDE axis. An understanding of the mechanisms underlying the reduced fluid secretion upon Dex treatment may lead to innovative strategies for the treatment of inflammation and salivary glands dysfunction during anesthesia without additional unwanted side effects.

## Materials and methods

### Reagents and DNA plasmids

Fura2-acetoxymethyl ester (Fura2-AM) and 2′,7′-bis-(carboxyethyl)-5-(and-6)-carboxyfluorescein (BCECF)-AM were purchased from Teflabs (Austin, TX). LPS from *P. gingivalis*, histamine, Dexmedetomidine hydrochloride (Dex), isoproterenol, carbamyl choline chloride (CCh), yohimbine (YOH), bumetanide (Bumeta), and all other chemicals not mentioned here were purchased from Sigma Aldrich (St. Louis, MO). Pluronic F-127 (20% in DMSO) and ZO-1 antibody were purchased from Invitrogen (Carlsbad, CA). PDE4 was purchased from Fabgennix (Frisco, TX), and IL-6 and caspase-1 antibodies were purchased from Abcam (Cambridge, MA). The α2_A_-adrenergic receptor antibody was purchased from Santacruz Inc. (Santa Cruz, CA). Collagenase P was purchased from Roche (Basel, Switzerland). The HA-tagged human AE2, pCMV6-AC-mKate-SLC26A6, and empty vectors were kindly provided by Dr. Shmuel Muallem (National Institutes of Health, Bethesda, MD, USA).

### Isolation of mouse submandibular glands cells and cultures of parotid sealed ducts and human salivary glands cell lines

All procedures for maintaining the mice and for the isolation of acini and ducts followed Gachon University guidelines and were approved by the Animal Care and Use Committee of Gachon University. Cultured, sealed parotid ducts from wild-type were prepared as described previously (Hong et al., 2015). SMG isolated from 20 to 25 g wild-type mice were washed and resuspended in physiological salt solution (PSS) A containing 140 mM NaCl, 10 mM glucose, 1 mM MgCl_2_, 5 mM KCl, 10 mM HEPES, and 1 mM CaCl_2_, pH 7.4, 0.02% STI, 0.1% sodium pyruvate, and 0.1% BSA (called PSA) and kept on ice until use. Briefly, the minced SMG was incubated in PSA containing 2.5 mg/10 ml collagenase P (Roche) for 8 min at 37°C. The digest was washed and resuspended with PSA, and kept on ice until use. For the culture of sealed ducts, C57BL/6N wild-type mice (20–25 g) were killed by cervical dislocation. The parotid glands were removed and injected with a digestion buffer consisting of serum-free DMEM, containing 50 U/ml collagenase, 400 U/ml hyaluronidase, 0.2 mg/ml soybean trypsin inhibitor (STI), and 2 mg/ml bovine serum albumin (BSA). The tissue was minced and incubated at 37°C for 30 min and then in fresh digestion buffer for a further 45 min. After a wash with DMEM containing 3% BSA and 0.2 mg/ml STI, ducts were microdissected from the partially digested tissue to remove acini or connective tissues. The ducts were cultured in DMEM supplemented with 10% fetal bovine serum at 37°C for 24 h before use. The HSG cells were purchased from American Type Culture Collection (Rockville, MD) and maintained in DMEM containing 10% FBS with antibiotics including 100 U/ml penicillin and 100 μg/ml streptomycin at 37°C in a cell culture incubator with 5% CO_2_/95% air atmosphere. When cells were 70~80% confluent, they were washed with PBS and dispersed with a 2 min trypsin/EDTA treatment before they were transferred to new culture dishes or glass coverslips-including dishes for later use.

### Measurement of fluid secretion by the sealed ducts

Ductal fluid secretion was measured by video microscopy as previously described (Yang et al., 2009; Hong et al., 2015). Briefly, the sealed ducts were transferred to a poly-L-lysine-coated perfusion chamber and perfused with HEPES- and then ${\text{HCO}}_{3}^{-}$-buffered media and stimulated with 5 μM forskolin in ${\text{HCO}}_{3}^{-}$-buffered media. Images were captured at 2 min intervals, obtained up to 40 min, and analyzed by calculating the lumen volume. Due to the variation in size between the microdissected ducts, a normalized procedure was used with the volume of the first image (V_0_) set as 1. Fluid secretion was represented as the ratio V_t_/V_0_, which was calculated from V_t_/V_0_ = (A_t_/A_0_)^3/2^.

### DNA transfection

Plasmid DNA transfection by Lipofectamine 2000 was followed by manufacturer's protocol (Invitrogen). Each plasmid DNA was diluted in 250 μl of Opti-Eagle's Minimum Essential Media (Opti-MEM™, Invitrogen), and 4 μl Lipofectamine 2000 was diluted in 250 μl of the same medium and incubated for 5 min at room temperature. The DNAs and Lipofectamine 2000 were mixed and after 25 min were added to the cell-cultured dish containing glass coverslip. After 4 h, the medium was replaced with a fresh DMEM medium containing FBS and the cells were used 24 h after the beginning of the transfection.

### Measurement of intracellular Ca^2+^ concentration

All procedures for mouse maintenance and for the isolation of submandibular and parotid acini and ducts followed Gachon University guidelines and were approved by the Animal Care and Use Committee of Gachon University. Salivary glands isolated from 25 to 30 g C57BL/6N mice were washed and re-suspended in PSA and kept on ice until use. Briefly, minced salivary gland cells were incubated in PSA containing 2.5 mg/10 ml collagenase P for 8 min at 37°C. The digest was washed with PSA, re-suspended in PSA, and kept on ice until use. Cells were transferred onto cover glasses and incubated with 4 μM Fura2-AM in the presence of 0.05% Pluronic F-127 for 30 min in PSS at room temperature, and then washed with PSS. Changes in intracellular Ca^2+^ concentration ([Ca^2+^]_i_) were determined by measuring the fluorescence intensities using dual excitation wavelengths of 340 and 380 nm and an emission wavelength of 510 nm. Results are presented as fluorescence ratios (Ratio = *F*_340/380_). The emitted fluorescence was monitored with a CCD camera (Photometrics, AZ) attached to an inverted microscope (Olympus, Japan) and analyzed with a MetaFluor system (Molecular Devices, PA). Fluorescence images were obtained at 1 s intervals and background fluorescence was subtracted from raw background signals at each wavelength.

### Measurement of Cl^−^-${\text{HCO}}_{3}^{-}$ exchange activity and ${\text{NH}}_{4}^{+}$ influx by pH_i_

Changes in pH_i_ were measured with BCECF-AM at dual excitation wavelengths of 440 and 495 nm and an emission wavelength of 530 nm. Isolated SMG cells attached onto coverslips were loaded in the chamber with 6 μM BCECF-AM in the presence of 0.05% Pluronic F-127 for 15 min at room temperature. After stabilizing the fluorescence, the cells were perfused with PSS for at least 5 min before measuring pH_i_ at 37°C. CBE activity was measured by incubating the cells with CO_2_-saturated ${\text{HCO}}_{3}^{-}$-buffered media to acidify the cytosol and initiated by perfusing the cells with Cl^−^-free ${\text{HCO}}_{3}^{-}$-buffered media. The emitted fluorescence was monitored with a CCD camera (Photometrics) attached to an inverted microscope (Olympus, Japan) and analyzed with a MetaFluor system (Molecular Devices). All fluorescence images were obtained at 1 s intervals and background fluorescence was subtracted from raw background signals at each wavelength. CBE activity was determined from the derivatives of the slopes from the first 30–45 s of pH_i_ increases in Cl^−^-free ${\text{HCO}}_{3}^{-}$-buffered media. ${\text{NH}}_{4}^{+}$ influx was measured from the rate of pH_i_ decrease induced by intracellular uptake of ${\text{NH}}_{4}^{+}$ as previously described (Evans and Turner, 1997; Worrell et al., 2004). Administration of NH_4_Cl in the extracellular solution induced the initial alkalization by diffusion of NH_3_, and then pH_i_ is decreased by ${\text{NH}}_{4}^{+}$ influx as a substitution of K^+^. The pH_i_ recovery rate in second phase provides ${\text{NH}}_{4}^{+}$ influx. The traces were normalized at time point of administration of NH_4_Cl. The acidified slope was calculated and represented as the ratio.

### Confocal imaging

Experiments were performed with isolated SMG acinar and ductal clusters. Isolated SMG acini and ducts were plated on glass coverslips for 5 min at room temperature prior to fixation with chilled methanol or 4% paraformaldehyde for 10 min. After fixation, immunostaining was performed as described previously (Lee et al., 2016) using 1:100 dilutions of the α2_A_-adrenergic receptor, PDE4, and ZO-1 antibodies. Briefly, the cells were incubated with the primary antibodies overnight at 4°C, then washed with 5% BSA/PBS. Remaining bound antibodies were detected with goat anti-rabbit immunoglobulin G (IgG) tagged with fluorescein isothiocyanate (FITC) (ZO-1) or rhodamine (PDE4) and then washed with PBS. Coverslips were mounted on glass slides with Fluoromount-G^TM^ (Electron Microscopy Sciences, Hatfield, PA) and analyzed using a LSM 700 Zeiss confocal microscope (Germany) with ZEN software. To determine the normalized intensity of PDE4 in each image, average intensity of PDE4 was divided by area. Images were collected from four to five separate preparations of acinar and ductal cells, and results are the averages from all experiments. The absence of a primary antibody was used as a negative control (NC). Fluorescent images were analyzed with a MetaMorph system (Molecular Devices).

### Semi-quantitative reverse transcription-polymerase chain reaction (semi-qRT-PCR)

Total RNA was extracted from isolated SMG and HSG cells using the Hybrid-RiboEx extraction system (Gentaur, Belgium) following the manufacturer's instructions, and amplified according to the manufacturer's protocol using TOPscript™ RT-PCR kit from Enzynomics (Daejeon, South Korea) and nested primers. The primers used are listed in Table 1. The PCR protocol comprised a denaturation step at 95°C for 5 min, followed by 35 cycles of 95°C for 1 min, an annealing step for 1 min, and an extension step at 72°C for 1 min, finally culminating with a final extension step at 72°C for 10 min. PCR products were electrophoresed on 1% agarose gels. Bands on agarose gels were visualized and acquired with a CCD camera and scanned using GelDoc^XR^ imaging system (Bio-Rad, CA). Intensities of PCR bands were analyzed with a MetaMorph system (Molecular Devices).

**Table 1:** **Information of primers**.

**Genes**	**Tm (°C)**	**Sequences (5′ → 3′)**
mPDE4A	55	(F) TTC AAG CTG CTG CAA GAA GA
		(R) TTC CTG AGG ACC TGG ATA CG
mPDE4B	55	(F) GAA CAA ATG GGG CCT TAA CA
		(R) TTG TCC AGG AGG AGA ACA CC
mPDE4C	57	(F) CAT GCT CAA CCG TGA GTT GT
		(R) TGG AAC GTC TTG AGG AGG TC
mPDE4D	57	(F) GGA GCT TGT CAC CTT CTT GG
		(R) GTG GGC TTT AAG TTG CTC CA
mGAPDH	55	(F) TTA GCC CCC CTG GCC AAG G
		(R) CTT ACT CCT TGG AGG CCA TG
mIL-6	63	(F) GAG GAT ACC ACT CCC AAC AGA CC
		(R) CTA TGG TAC TCC AGA AGA CCA GAG
mNOX2	57	(F) GTG TTG CTC GAC AAG GAT TC
		(R) CTC CGA ATG GTT TTG GTA GAG
mNOX4	58	(F) CAG CTT CTA CCT ACG CAA TAA G
		(R) GGA AAT GAG CTT GGA ACT TGG
hPDE4	62	(F) GTT GAG ACG AAG AAG GTG ACC AG
		(R) GTC GGC CCA TGT TTC CCA CAA TG
hGAPDH	52	(F) GTC GGA GTC AAC GGA TT
		(R) GCC ATG GGT GGA ATC ATA

### Western blot

SMG cells were isolated and stimulated with the indicated components for 1 h. Cell lysates were prepared in lysis buffer (containing [mM] 20 Tris, 150 NaCl, 2 EDTA, 1% Triton X-100, and a protease inhibitor mixture) and treated as previously described (Lee et al., 2016). Briefly, proteins were denatured via incubation in SDS sample buffer at 37°C for 30 min. The 30 μg of denatured protein samples were subjected to SDS-PAGE and transferred to methanol-soaked polyvinylidene difluoride (PVDF) membranes. Transferred proteins on PVDF membranes were visualized with PDE4 (Fabgennix), α2_A_-adrenergic receptor (Santacruz), IL-6 and caspase-1 (Abcam), and β-actin (Sigma) antibodies by enhanced luminescence solution (Thermo Scientific).

### Measurement of cAMP concentrations

The cAMP concentration for each sample was determined by cAMP EIA kit (Cayman Chemical Company, Ann Arbor, MI) according to the manufacturer's instructions. Briefly, absorbance wavelength for assay was 405 nm, and cAMP concentration was determined by calculation based on a standard curve. Protein concentrations were measured by Quick Start Bradford Assay (Bio-Rad, Hercules, CA). Each cAMP values were normalized to amount of protein for each samples.

### Statistical analysis

Results from the indicated number of experiments were expressed as mean ± SEM. Differences between means were considered statistically significant when *P* < 0.01 (*) or *P* < 0.05 (#).

## Results

### Effect of Dex on CBE activity in isolated mouse SMG cells and fluid secretion in sealed parotid ducts

**Figure 1:**
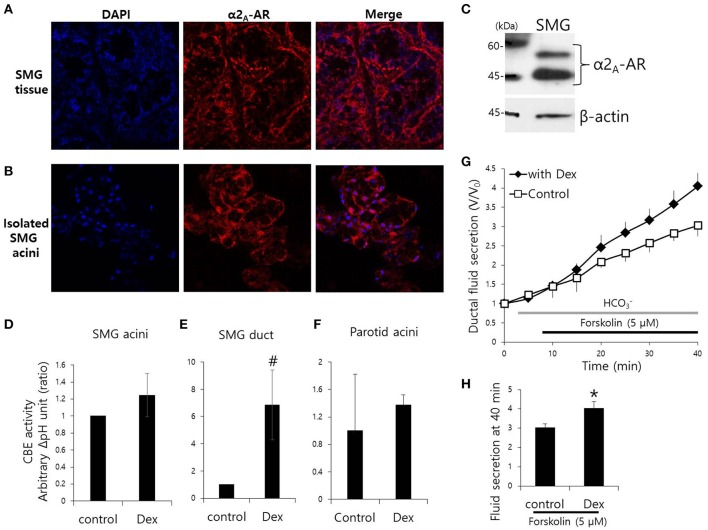
**Effect of Dex on CBE activity in isolated mouse SMG cells and fluid secretion in sealed parotid ducts. (A,B)** Localization of α2_A_-adrenergic receptor (AR) in SMG tissue and isolated SMG acini. **(C)** Protein expression α2_A_-AR in SMG. CBE activity was determined by measuring changes in pH_i_ in SMG acini **(D)**, SMG ducts **(E)**, and parotid acini **(F)** with and without 100 ng/ml Dex. The slope of pH_i_ measured CBE activity in the absence of Cl^−^ at the beginning of time course (30–45 s), and height to reach the point of maximum pH_i_ from the minimum point. Bars represent the mean ± SEM (*n* = 4, ^*^p < 0.01, #*p* < 0.05). **(G)** Sealed parotid ducts were isolated and used to measure fluid secretion in response to stimulation with 5 μM forskolin in the absence (control, open square) and presence of 100 ng/ml Dex (closed rhombus) (*n* = 4, ^*^p < 0.01). **(H)** The mean ± SEM at the 40 min secretion time point shows an increase in basal secretion upon Dex treatment.

Mouse SMG tissue and isolated SMG cells were stained with α2_A_-adrenoceptor antibody (Figures 1A,B). Protein expression of α2_A_-adrenoceptor in SMG was evaluated by western blot analysis (Figure 1C). We have demonstrated for the first that the α2_A_-adrenoceptor is expressed in the basolateral membrane of SMG cells. Then, to determine the effects of Dex on the modulation of ion transporter activity, we measured CBE activity in parotid, SMG acini, and SMG ductal cells. CBE activity was evaluated by measuring the change in pH_i_ induced by acute Cl^−^ removal and subsequent addition of Cl^−^ in the presence or absence of Dex. Removal of Cl^−^ in the perfused solution induced intracellular alkalization. The slope of the change in pH_i_ was measured using Cl^−^-free ${\text{HCO}}_{3}^{-}$-buffered solution. No changes in CBE activity were found in SMG and parotid acini cells upon Dex treatment (Figures 1D–F), CBE activity in SMG ductal cells was markedly increased upon acute Dex treatment (Figure 1E). Our previous study suggested that CBE is essential for ductal ${\text{HCO}}_{3}^{-}$ secretion in the salivary glands (Hong et al., 2015). The clinical side effects of Dex such as hyposecretion raised the question of whether Dex participates in modulation of ${\text{HCO}}_{3}^{-}$ transporters. To address this question, we measured ductal fluid secretion using the sealed parotid ductal *ex vivo* system. Interestingly, in contrast to the hyposecretion effect observed clinically (Marks et al., 2010), Dex increased forskolin-stimulated parotid ductal fluid secretion (Figure 1G). Dex-stimulated fluid secretion at 40 min was 33.5 ± 11.2% higher than in the control (Figure 1H). However, long-lasting Dex treatment for 2 h showed no changes in ductal fluid secretion compared to control (data not shown). These data suggest that CBE activity of ductal cells and ductal fluid secretion were activated by acute treatment with Dex.

### Effect of Dex on ${\text{NH}}_{4}^{+}$ influx in mouse isolated SMG and HSG cells

**Figure 2:**
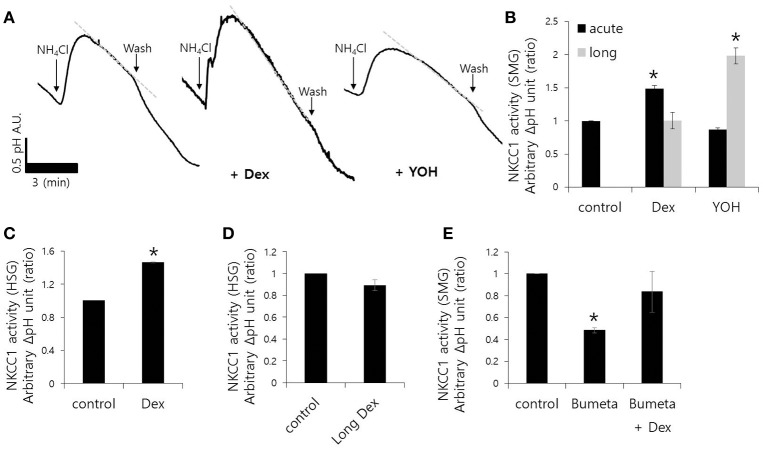
**Effect of Dex on ${\text{NH}}_{4}^{+}$ influx in mouse isolated SMG and HSG cells**. ${\text{NH}}_{4}^{+}$ influx was assessed by measuring changes in pH_i_ in SMG acini with and without 100 ng/ml Dex. The rate of ${\text{NH}}_{4}^{+}$ influx (gray dotted line) was determined from the pH_i_ recovery rate in the second phase after 20 mM NH_4_Cl pulse. Bars represent the mean ± SEM (*n* = 5). **(A)** Traces of the NH_4_Cl pulses used to measure ${\text{NH}}_{4}^{+}$ influx with 100 ng/ml Dex or 10 μM YOH in isolated SMG acini. **(B)** Analysis of ${\text{NH}}_{4}^{+}$ influx in isolated SMG acini. Bars represent the mean ± SEM (*n* = 4, ^*^p < 0.01). Analysis of ${\text{NH}}_{4}^{+}$ influx in HSG cells **(C,D)** and SMG acini **(E)** in the presence of the indicated components including 100 μM Bumetanide (Bumeta). Bars represent the mean ± SEM (*n* = 4, ^*^p < 0.01).

Fluid secretion is driven by the luminal Cl^−^ efflux and the basolateral Cl^−^ influx that occurs across the plasma membrane (Lee et al., 2012). The basolateral Cl^−^ influx is mediated by NKCC1. We measured ${\text{NH}}_{4}^{+}$ influx by measuring the change in pH_i_ during 20 mM NH_4_Cl pulse with and without Dex. ${\text{NH}}_{4}^{+}$ influx increased in the presence of Dex in isolated SMG acini, however, ${\text{NH}}_{4}^{+}$ influx decreased in the presence of the α2-adreneric antagonist 10 μM yohimbine (YOH; Figures 2A,B). A longer Dex treatment revealed no effect on ${\text{NH}}_{4}^{+}$ influx, whereas, long-lasting YOH treatment (2 h) increased ${\text{NH}}_{4}^{+}$ influx (Figure 2B). HSG cells were also used to evaluate the long-lasting Dex treatment on ${\text{NH}}_{4}^{+}$ influx. ${\text{NH}}_{4}^{+}$ influx was increased in HSG cells upon acute Dex treatment, but was partially inhibited with long- lasting Dex treatment for 2 h (Figures 2C,D). Part of ${\text{NH}}_{4}^{+}$ influx is mediated by NKCC1 (Blanco et al., 2013). Bumetanide (Bumeta) is an inhibitor of NKCC1. The minor effects of NH4^+^ entry observed with 10 μM Bumeta (data now shown). The 100 μM Bumeta showed 52% inhibition of NH4^+^ entry. Dex treatment partially restored the Bumeta-associated ${\text{NH}}_{4}^{+}$ entry (Figure 2E). These data suggest that NKCC1-independent ${\text{NH}}_{4}^{+}$ influx was activated by acute Dex treatment with Dex, whereas, long-lasting Dex treatment did not inhibit ${\text{NH}}_{4}^{+}$ entry.

### Long-lasting Dex treatment on CBE activity in mouse isolated SMG and HSG cells

**Figure 3:**
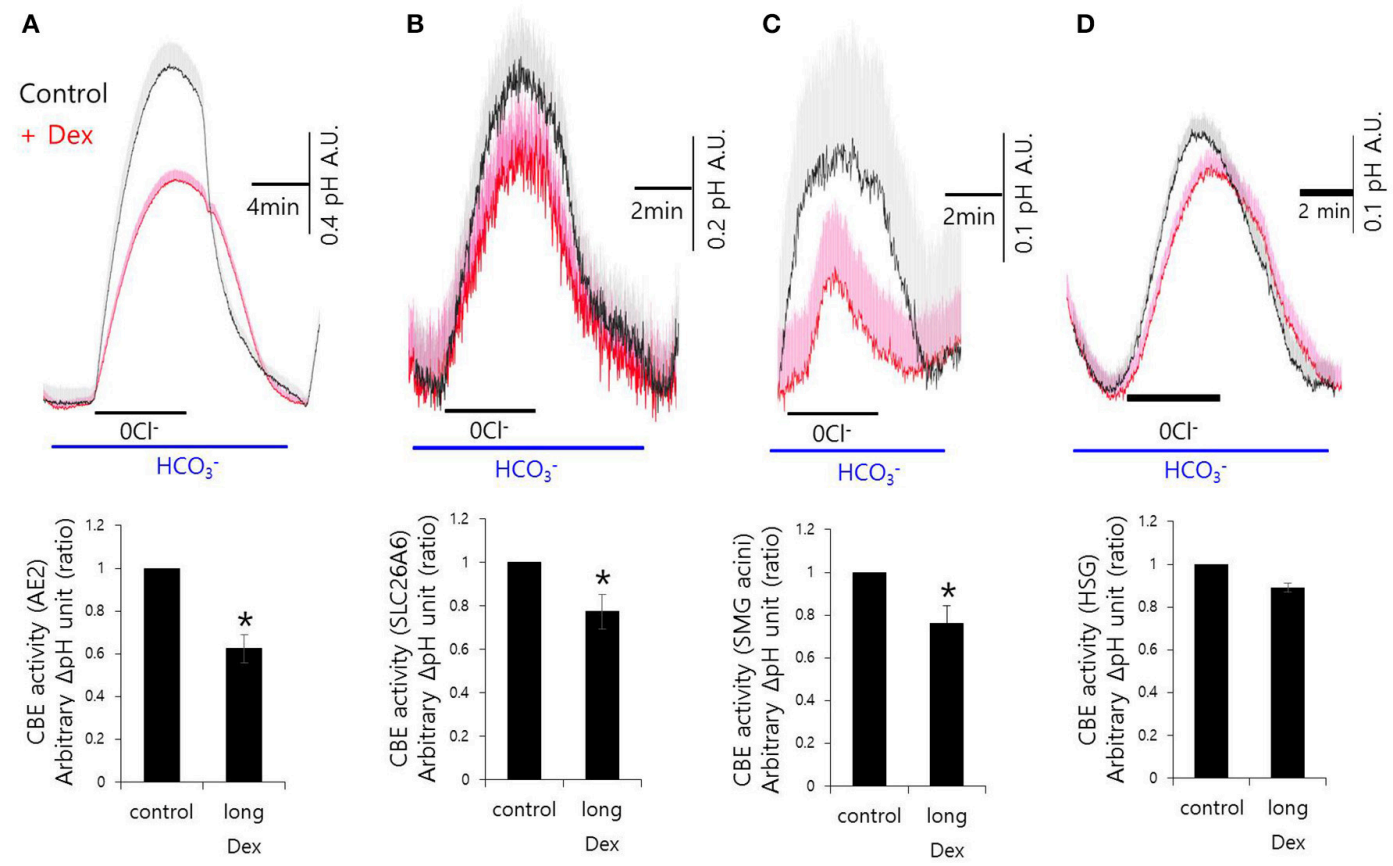
**Long-lasting Dex treatment on CBE activity in in mouse isolated SMG and HSG cells**. SMG and HSG cells were treated with Dex for 2 h. CBE activity was assessed by measuring changes in pH_i_ in AE2-transfected cells **(A)**, in SLC26A6-transfected cells **(B)**, in SMG acini **(C)**, and in HSG cells **(D)** with and without 100 ng/ml Dex. Bars show the mean ± SEM (*n* = 4, ^*^p < 0.01).

Although, Dex stimulates CBE activity and ${\text{NH}}_{4}^{+}$ entry, it remains unclear how Dex induces fluid hyposecretion. We hypothesized that the Dex effect was time-dependent, thus to evaluate the long-lasting treatment with Dex, we measured CBE activity in cells that were incubated with Dex for 2 h. To avoid isolated SMG cells instability during the prolonged treatment period, we employed a CBE overexpression system that included basolateral anion exchanger 2 (AE2) and luminal solute carrier 26 family SLC26A6 (Figures 3A,B). CBE activity in isolated SMG acini and in HSG cells were inhibited by long-lasting Dex treatment (Figures 3C,D), suggesting that Dex-induced inhibition of ion transporters plays a role in fluid hyposecretion.

### Differential effects of Dex on neurotransmitter inputs and inflammatory mediator-induced intracellular calcium signaling in mouse SMG cells

**Figure 4:**
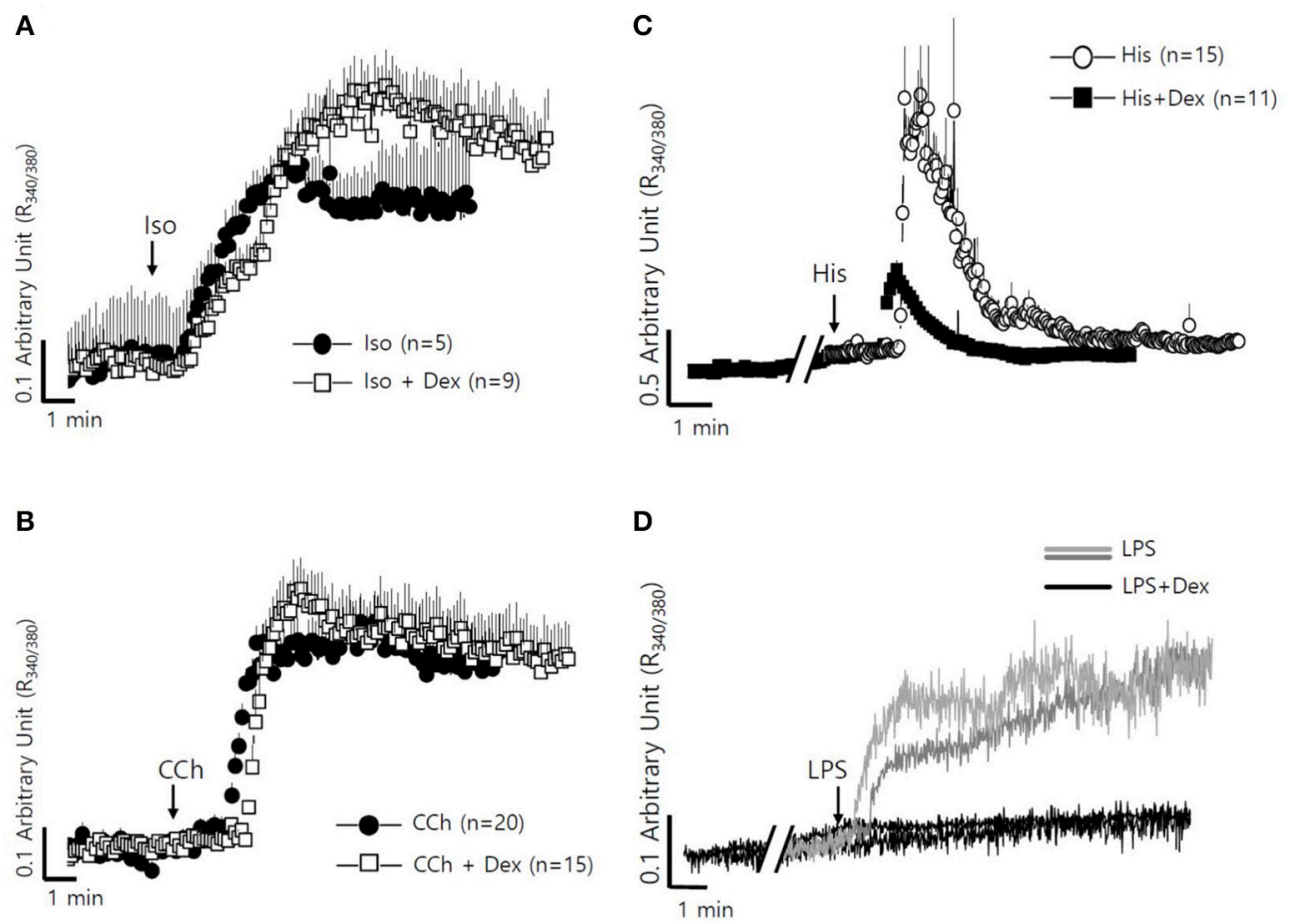
**Differential effects of Dex on neurotransmitter inputs and inflammatory mediator-induced intracellular calcium signaling in mouse SMG cells. (A)** Changes in [Ca^2+^]_i_ induced by 100 μM isoproterenol without (closed circle) and with pre-treatment with 100 ng/ml Dex (open square). All of the traces were averaged. **(B)** Changes in [Ca^2+^]_i_ induced by 10 μM carbachol without (closed circle) and with pre-treatment with 100 ng/ml Dex (open square). All of the traces were averaged. **(C)** Changes in [Ca^2+^]_i_ induced by 100 nM histamine without (closed circle) and with pre-treatment with 100 ng/ml Dex (closed square). All of the traces were averaged. **(D)** Changes in [Ca^2+^]_i_ induced by 20 μg/ml LPS in ducts and in acini without (gray line for ducts and light gray line for acini) and with (black line) pre-treatment with 100 ng/ml Dex. A single trace is shown. Arrows indicate when stimuli were applied to the cells.

The fluid secretion function of the salivary glands is regulated by numerous components, including neurotransmitters that modulate spatial and temporal Ca^2+^ signaling (Lee et al., 2016). The role of Dex on cholinergic/adrenergic inputs-induced intracellular Ca^2+^ concentration ([Ca^2+^]_i_) increases was assessed in SMG cells. Dex did not alter [Ca^2+^]_i_ signals in SMG cells (data not shown). SMG cells were stimulated with the β-adrenergic agonist isoproterenol (Iso) and the cholinergic agonist carbamyl choline chloride (CCh) with and without Dex (Figures 4A,B). No effect of Dex stimulation was observed in CCh- and Iso-induced [Ca^2+^]_i_ signals. To evaluate whether the regulatory effect of Dex in isolated SMG cells is mediated by inflammatory mediators, such as histamine and LPS, histamine-, and LPS-induced [Ca^2+^]_i_ measurements were performed with and without Dex. Inflammatory mediators-induced [Ca^2+^]_i_ signals were inhibited by Dex (Figures 4C,D), thus Dex appears to play a role in inflammatory mediators-induced Ca^2+^ signaling.

### Effect of Dex on PDE4 expression

**Figure 5:**
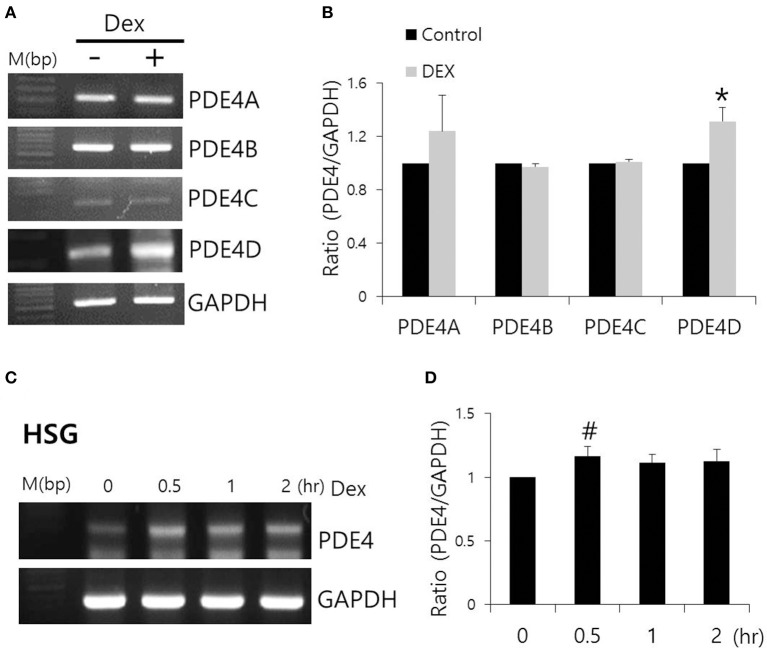
**Effect of Dex on PDE4 mRNA expression. (A)** The mRNA expression of *PDE4A–D* subfamily members with and without 100 ng/ml Dex in SMG cells. **(B)** Results are expressed as fold expression relative to the control and mean ± SEM (*n* = 3, ^*^p < 0.01). **(C,D)** Time-dependent *PDE4* mRNA expression with and without 100 ng/ml Dex in HSG cells (*n* = 3, #*p* < 0.05).

**Figure 6:**
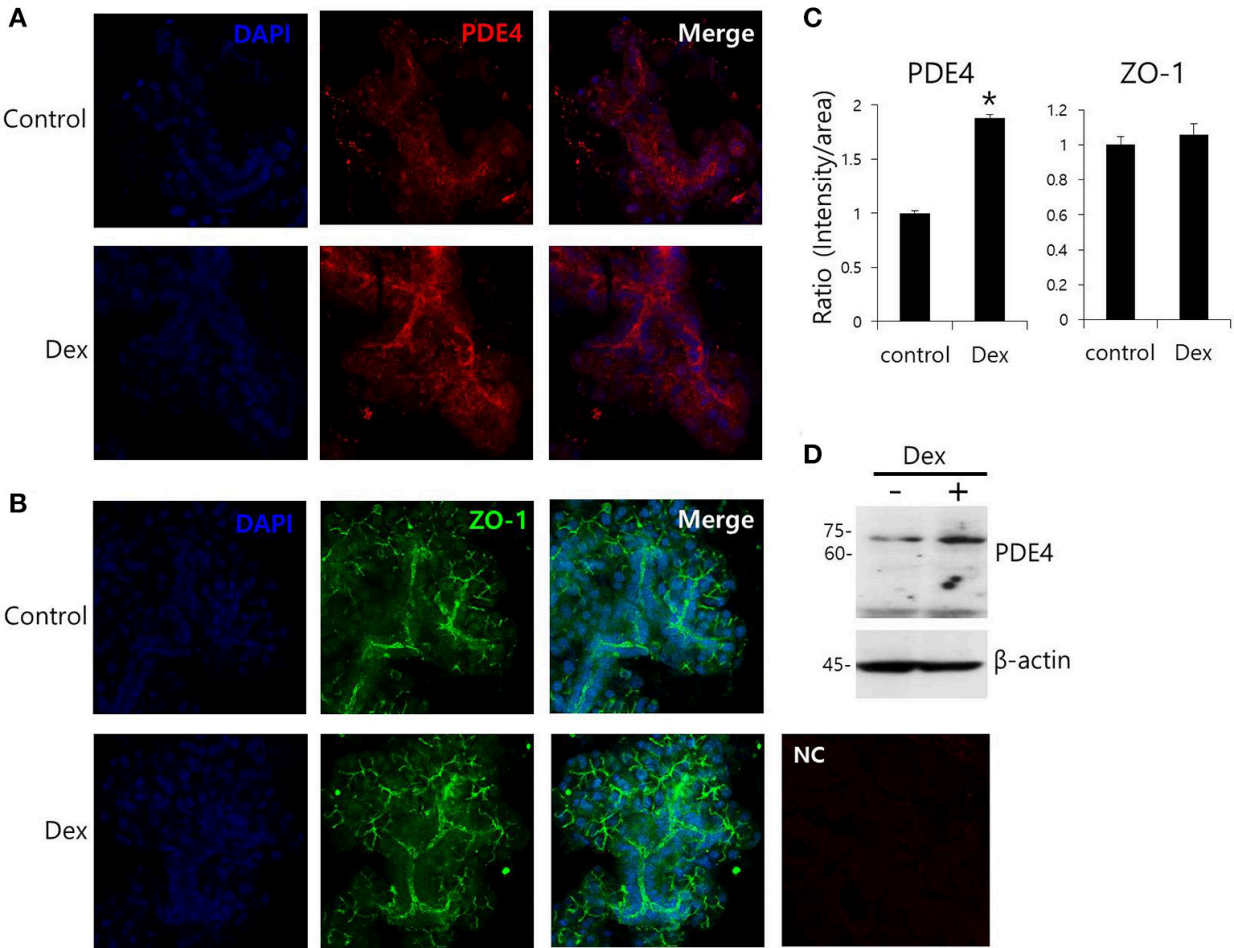
**Effect of Dex on PDE4 expression. (A,B)** Immunofluorescence staining of PDE4 (red) and ZO-1 (green) in isolated SMG cells. **(C)** Relative intensities of PDE4 and ZO-1 staining divided by area. Bars represent the mean ± SEM (*n* = 3, ^*^p < 0.01). NC, negative control. **(D)** PDE4 and β-actin protein expressions with and without 100 ng/ml Dex in SMG cells.

Dex has been linked to post-cAMP activation cellular events in the renal collecting duct (Rouch et al., 1997). PDE4, which consists of four subfamilies, PDE4A-4D, is a well-characterized cAMP-specific enzyme that is expressed in SMG cells (Lee et al., 2016). To determine the effect of Dex on PDE4 expression in SMG cells, we performed semi-qRT-PCR using cells treated with Dex for 2 h. *PDE4D* mRNA expression increased in the presence of Dex (Figures 5A,B). Dex-induced time-dependent increase in *PDE4* mRNA expression was also seen in HSG cells (Figures 5C,D). We confirmed this Dex-induced stimulatory effect by immunostaining isolated SMG cells with PDE4 antibody (Figures 6A,C). As a specificity control, expression of the tight junction marker ZO-1 did not change in the presence of Dex (Figures 6B,C). We also found that PDE4 was dominantly expressed in the luminal membrane of SMG cells and that increased PDE4 expression was observed in Dex-treated SMG cells. We confirmed the increase in PDE4 protein expression in the presence of Dex for 2 h by an enhancement in the 65-kDa band that corresponds to PDE4 (Figure 6D).

### Dex-induced inhibition of LPS-induced inflammatory cytokine expression and cAMP concentration

**Figure 7:**
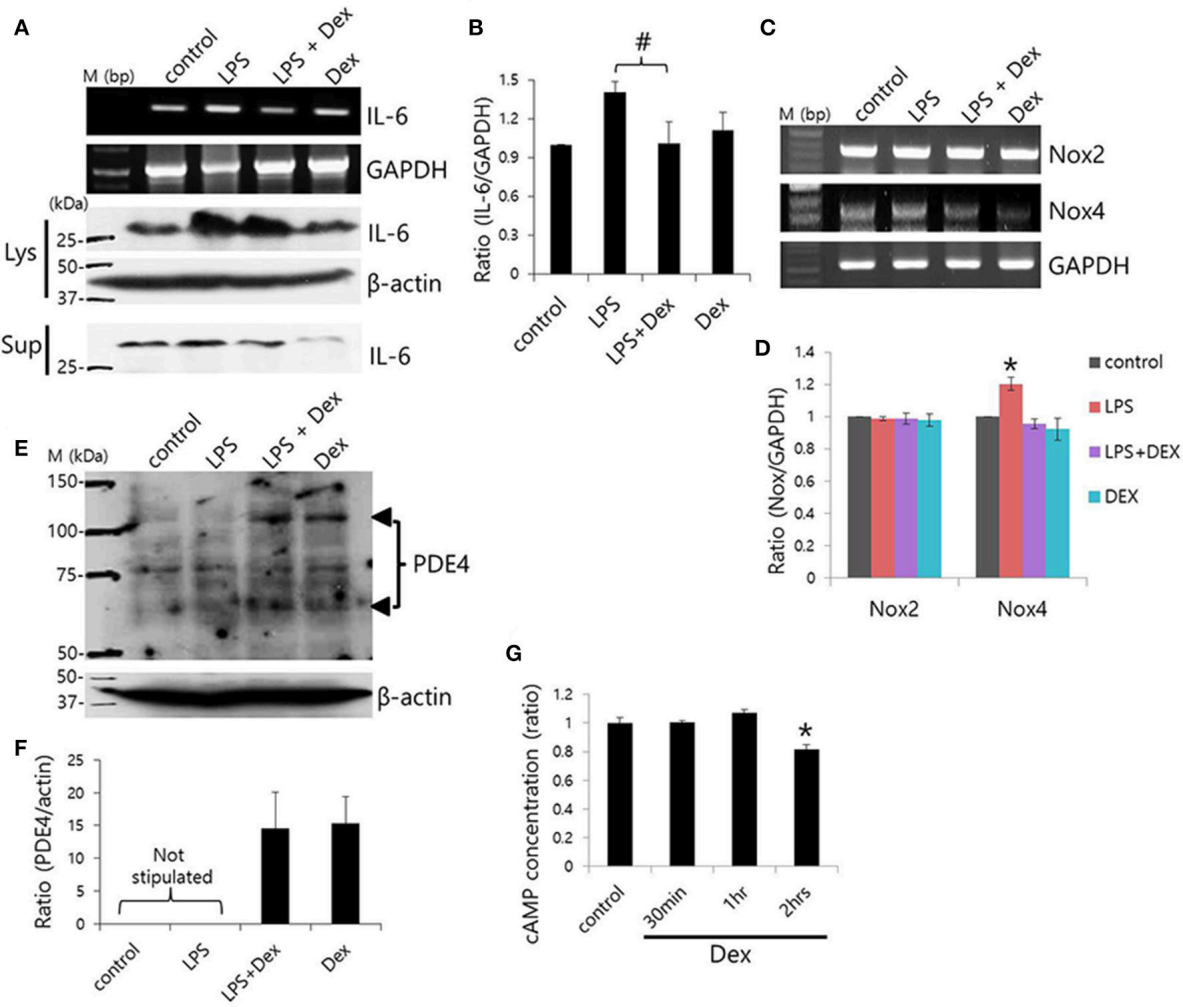
**Dex-induced inhibition of LPS-induced inflammatory cytokine expression and cAMP concentration**. **(A)** SMG cells were stimulated with LPS, treated with 100 ng/ml Dex, and then IL-6 mRNA and protein expression were measured in cell lysates (Lys) and cell supernatants (Sup). **(B)** Bars represent the mean ± SEM (*n* = 4, #*p* < 0.05). **(C)** LPS-induced *Nox2* and *Nox4* mRNA expression in the presence of 100 ng/ml Dex. **(D)** Analysis of *Nox2* and *Nox4* mRNA expression in the presence of the indicated components (*n* = 5, ^*^p < 0.01). **(E)** PDE4 protein expression in LPS-stimulated SMG cells. The arrowheads indicate PDE4A and PDE4D bands at 65 and 102 kDa, respectively. **(F)** Analysis of PDE4 expression. Bars show the mean ± SEM (*n* = 4). **(G)** cAMP ratios in Dex-treated whole SMG cells (*n* = 4, ^*^p < 0.01).

To evaluate the anti-inflammatory role of Dex on SMG cells, cells were stimulated with LPS from *P. gingivalis*, and IL-6 expression was measured. Previously, we found that SMG cells express TLR4, which is involved in LPS signaling (Lee et al., 2016). Dex inhibited LPS-induced *IL-6* mRNA expression (#*p* < 0.05), however, cytosolic and secreted IL-6 protein levels showed no statistical difference (*p* < 0.1; Figures 7A,B).

NADPH oxidases (Nox) are a major source of reactive oxygen species (ROS; Vieceli Dalla Sega et al., 2014), and LPS-induced oxidative stress was found to increase production of ROS through Nox4 (Ngkelo et al., 2012). Normal salivary glands cells express Nox2 and Nox4 (Tateishi et al., 2008). We confirmed that SMG cells expressed *Nox2* and *Nox4* mRNA and that Dex attenuated *Nox4*, but not *Nox2* mRNA expression in SMG cells (Figures 7C,D). To further examine the anti-inflammatory role of Dex, cells were stimulated with LPS and exposed to Dex. The presence of enhanced bands at 65 and 102 kDa, which correspond to PDE4A and PDE4D, respectively, in a Western blot revealed increased PDE4 expression in LPS-stimulated cells in the presence of Dex (Figures 7E,F). We obtained the enhanced bands at 65 and 102 kDa, respectively in the presence of Dex. To confirm the effects of Dex on cAMP concentration, isolated SMG cells were treated with Dex for various times (Figure 7G). The activation of α2 adrenoceptors activates G_i_ protein, which inhibits adenylate cyclase. Therefore, the activation of α2 adrenoceptors results in lower cAMP levels. Analysis of cAMP concentration relative to time of Dex treatment indicated that 2 h of Dex treatment reduced cAMP levels in isolated SMG cells.

## Discussion

The major novel finding of the present study was that α2-adrenoceptors are expressed in the basolateral membrane of mouse SMG cells and that the selective α2-adrenoceptor agonist Dex acutely increased ductal CBE activity and NKCC1-independent ${\text{NH}}_{4}^{+}$ entry, as well as increased fluid secretion in primary isolated SMG acini and ductal cells. Long-lasting treatment (2 h) with Dex inhibited CBE activity but not ${\text{NH}}_{4}^{+}$ entry. Reduced function of transporters caused by the increased expression of PDE4, which may be involved in reduced cAMP level in SMG. Recent evidence indicated that the α2-adrenoceptor agonist Dex has anti-inflammatory properties in various tissues (Peng et al., 2013; Tanabe et al., 2014; Yang and Hong, 2015). In this study, we also demonstrated an anti-inflammatory role for Dex in which Dex inhibits IL-6 mRNA and protein expression and *Nox4* mRNA expression in LPS-stimulated SMG cells.

A previous study demonstrated that the administration of Dex induced low systolic and diastolic blood pressure even though an initial blood pressure increase was observed (Karhuvaara et al., 1991). Previously, we found that the PDE4 inhibitor rolipram regulates intracellular cAMP levels and inhibits inflammatory signaling in the salivary glands and that apical localization of PDE4 may be involved in the activation of cAMP-dependent secretion (Lee et al., 2016). Inhibition of PDE prevents cAMP breakdown, increases intracellular cAMP concentrations, and may lead to cardiac stimulation. However, Dex-mediated PDE-sensitivity, as well as the differential roles and expression patterns of each PDE subfamily member should be further investigated in cardiac tissue and in the salivary glands.

**Figure 8:**
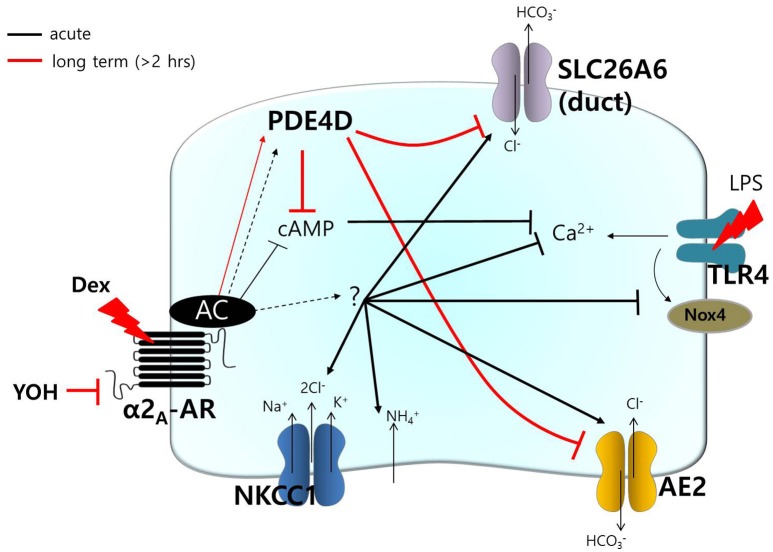
**Schematic model of Dex-modulated signaling**. Dex, dexmedetomidine; α2_A_-AR, α2_A_-adrenoceptor; NKCC1, Na^+^-K^+^-2Cl^−^ cotransporter1; AE2, anion exchanger2; SLC26A6, solute carrier 26 family A6; Nox4, NADPH oxidase 4; LPS, lipopolysaccharide; TLR4, toll-like receptor 4; PDE, phosphodiesterase; YOH, yohimbine. Acute (black) and long-lasting (red) treatment with Dex.

${\text{HCO}}_{3}^{-}$ transporters, including AE2 and SLC26A6, have been shown to be required for cAMP-stimulated anion secretion (Gawenis et al., 2010; Ahuja et al., 2014). Apical cAMP-associated ion transporters localize to the PDE4-rich environment following long Dex treatment, and this may induce impaired saliva secretion. In the salivary glands, PDE activity varies depending on intracellular cAMP concentrations due to salivary stimulation during the tissue development (Tanaka et al., 2002). Although, the cellular distributions and expression levels of PDE family members in the salivary glands remain to be elucidated, the long Dex-induced increase in PDE4D expression led to a reduction in the function of the cAMP-dependent transporters NKCC in acini and the Cl^−^/${\text{HCO}}_{3}^{-}$ exchanger in ductal cells. To our knowledge, this is the first demonstration of long-lasting treatment with Dex-induced modulation of PDE4 expression. It will be of particular interest to determine whether acute stimulation of Dex requires signaling molecule to activate transporters including AE2 and SLC26A6, activates other Cl^−^ transporters (e.g., cystic fibrosis transmembrane conductance regulator), or activates directly (Figure 8). The prediction of this model is that Dex may possess the dominant effect on Cl^−^ movement.

PDE4 functionally associates with the α2-adrenoceptor by modulating intracellular levels of cAMP in noradrenergic neurons (Robichaud et al., 2002). In this study, we provided direct evidence that Dex modulates PDE4D expression. PDE4 activation has been found to be involved in a broad spectrum of cellular events, for example, depletion of Nox4 decreases the cAMP concentration and aquaporin 2 (AQP2) expression in a PDE4-dependent manner in renal collecting duct principal cells (Féraille et al., 2014). As shown in Figure 7C, administration of Dex also inhibits Nox4 expression in SMG cells. As numerous physiological roles have been attributed to Nox4 in various tissues, a Nox4 deficiency as a result of long-lasting treatment with Dex could associate with kidney and salivary glands function, including AQP expression. PDE4 activation also attenuates cardiomyocyte hypertrophy by inhibiting nuclear PKA activity (Fan Chung, 2006).

The side effects of PDE4 inhibitors have been linked to PDE4-related emesis (Robichaud et al., 2002). Although, it has been difficult to define the role of PDE4 in emesis, activation of the α2-adrenoceptor by Dex leads to enhanced expression of PDE4D in the salivary glands.

This study suggests that activation of the α2-adrenoceptor and Dex-mediated regulation of PDE4D play roles in salivary signaling. Moreover, the cAMP/PDE4D axis is associated with the microenvironment of the transporters in the salivary glands. Thus, clearer understanding of PDE4-dependent/independent cellular signaling in the salivary glands could prevent side effects, such as fluid hyposecretion after operations and could prevent choking on saliva during anesthesia.

## Author contributions

For correspondence, KS and JH contributed equally to this work. JH, JL, and MJ performed and interpreted experiments; KP, CP, and KS conceived and directed the studies; and JH wrote the manuscript with contribution by all authors.

### Conflict of interest statement

The authors declare that the research was conducted in the absence of any commercial or financial relationships that could be construed as a potential conflict of interest.
